# Impact of Photoselective Nets on Phenolic Composition and Antioxidant Capacity in Different Apple Cultivars Under the Same Edaphoclimatic Conditions

**DOI:** 10.3390/molecules30091995

**Published:** 2025-04-30

**Authors:** João David Teixeira, Miguel Leão de Sousa, Sílvia Cruz Barros, Pier Parpot, Carina Almeida, Ana Sanches Silva

**Affiliations:** 1 National Institute for Agrarian and Veterinary Research (INIAV), I.P., Rua dos Lágidos, Lugar da Madalena Vairão, 4485-655 Vila do Conde, Portugal; david.teixeira@iniav.pt (J.D.T.); silvia.barros@iniav.pt (S.C.B.); carina.almeida@iniav.pt (C.A.); 2 Center for Study in Animal Science (CECA), Institute of Sciences, Technologies and Agroenvironment of the University of Porto (ICETA), University of Porto, Praça Coronel Pacheco, 4050-453 Porto, Portugal; 3 Chemistry Centre of the University of Minho (CQ-UM), University of Minho, 4710-057 Braga, Portugal; parpot@quimica.uminho.pt; 4 National Institute for Agrarian and Veterinary Research (INIAV), I.P., Estrada de Leiria, 2460-059 Alcobaça, Portugal; miguel.leao@iniav.pt; 5 Centre of Biological Engineering (CEB), University of Minho, 4710-057 Braga, Portugal; 6 LEPABE–Laboratory for Process Engineering, Environment, Biotechnology and Energy, Facultyof Engineering, University of Porto, Rua Dr. Roberto Frias, 4200-465 Porto, Portugal; 7 AliCE–Associate Laboratory in Chemical Engineering, Faculty of Engineering, University of Porto, Rua Dr. Roberto Frias, 4200-465 Porto, Portugal; 8 University of Coimbra, Faculty of Pharmacy, Polo III, Azinhaga de SantaComba, 3000-548 Coimbra, Portugal; 9 Associate Laboratory for Animal and Veterinary Sciences (Al4AnimalS), 1300-477 Lisbon, Portugal

**Keywords:** antioxidants, apples, phenolics, photoselective nets, UHPLC-ToF-MS

## Abstract

Phenolic compounds in apples provide significant health benefits, including antioxidant, and anti-inflammatory properties. The phenolic profile and content in apples are influenced by genetics, environmental factors, and agricultural practices. Photoselective nets, which are designed to filter specific wavelengths of light, might impact fruit quality and phenolic content. This study aimed to assess the effects of photoselective nets on the antioxidant capacity and phenolic composition of three different apple cultivars grown under the same edaphoclimatic conditions. Five nets were selected. Fruits grown under the nets were compared with unprotected fruits. Antioxidant capacity was evaluated, and phenolic profiles were established by Ultra-High Performance Liquid Chromatography coupled with Time of Flight–Mass Spectrometry (UHPLC-ToF-MS). The results demonstrate a significant impact of the nets on the phenolic composition and antioxidant activities of apples. Different net colors had distinct effects on the accumulation of phenolic compounds, with some nets increasing flavonoid production and others reducing the levels of important phenolic acids. The gray and IRIDIUM^®^ Red nets enhanced the production of quercetin and its derivatives, while chlorogenic acid showed a general decline under net-covered conditions, indicating a possible dependence on direct sunlight. The responses were also cultivar-dependent, with *Gala redlum* apples showing the largest reductions in phenolic compounds when protected by nets. Antioxidant assays also confirmed that the nets influenced the antioxidant potential of apples in a cultivar-dependent manner. These findings suggest that the retention of bioactive compounds in fruits might be strategically managed by selecting appropriate net materials for specific cultivars.

## 1. Introduction

Phenolic compounds are a large group of secondary metabolites of fruits and vegetables of great interest because, since they act as reducing agents, they have various beneficial bioactivities for human health, including antioxidant [1,2], anti-inflammatory [3,4], and neuroprotective [5] properties, as well as action against diseases such as cancer [6]. The inclusion of these compounds in the human diet may help explain the health benefits associated with the consumption of fruits and vegetables [7].

Apples (*Malus domestica*) are a highly nutritious fruit, providing essential vitamins, minerals, and dietary fiber and are also rich in bioactive compounds, with phenolic compounds being particularly abundant [8]. These compounds play a crucial role in the fruit’s antioxidant capacity, flavor, and color, the latter of which is largely influenced by anthocyanins [9], the main pigments responsible for red hues. It is one of the most produced fruits worldwide (more than 95 million tons) [10], coming only after bananas and watermelons. More than 50% of the world’s production is located in China, and more than 7500 cultivars have been identified throughout the world, each exhibiting distinct characteristics, including variations in size, shape, color, texture, flavor, and nutritional composition [11]. The heterogeneity of apple cultivars (Malus domestica) and their phenolic composition are mainly determined by genetics, environmental conditions, and agronomic techniques, as well as the stage of ripeness, post-harvest handling, storage conditions, and processing methods, all of which influence the concentration, distribution, and stability of these bioactive compounds [12]. This great diversity, seen across apple cultivars and being caused by variations in gene expression, controls the formation of phenolic compounds and sugar content, among other characteristics. Additionally, edaphoclimatic conditions, such as temperature, solar exposure, rainfall, and soil type, have an impact on this diversity. For example, variations in temperature can alter the accumulation of anthocyanins [13], which can change the color of the fruit, while exposure to sunlight is essential for the synthesis of phenolic compounds [14].

A novel approach in fruit farming called photoselective netting is being used, in addition to neutral color nets, to promote changes, not only in the amount of incident radiation (acting according to the shading factor) but also in light quality, changing the environment in which fruit grows. These nets, which are mainly designed to regulate the spectrum of light that plants receive, promote different physiological responses, since plants perceive these light signals through specialized photoreceptors, including phytochromes, which absorb light in the red (R) and far-red (FR) ranges; cryptochromes, which respond to blue/UV-A light; and phototropins. This, among other physiological processes, can occur in the formation of secondary metabolites such as phenolic compounds, being a consequence of the materials that selectively filter particular wavelengths of solar radiation [15]. In addition to affecting the light spectrum and direct and diffuse radiation, photoselective nets help regulate the temperature and microclimate around plants [16]. By reducing excessive thermal radiation, and hence decreasing heat stress and dehydration, overall better fruit quality can be achieved. This was highlighted by Li et al. [16], who showed that tannin, flavanol, and total aromatic volatile contents in wine increased by 95%, 40%, and 10%, respectively.

Depending on the color and type of netting used, nets can induce different physiological and biochemical responses in the plant, influencing fruit characteristics such as color, texture, and nutritional value. The main types of nets are black and white; however, nets with different chromatic particles have also been used [17]. Several studies have shown that photoselective nets are an efficient way to control fruit quality. According to research in apple orchards, white nets yielded heavier fruits (202.37 g), whereas blue and red nets were less efficient (182.75 and around 190 g, respectively). Yellow nets initially increased fruit growth, but this trend was not maintained throughout the season, resulting in a reduced final fruit weight [17]. The impacts of photoselective nets extend to fruit chemical composition, such as phenolic content and volatile compound content. In blueberries [15], black nets greatly increased the total phenolic content and primary metabolites, making them the most effective at increasing these compounds. However, their impact on volatile substances was inconsistent throughout the study, indicating the importance of environmental variability. In grapes [16], pearl nets decreased the content of flavanol (12.41 compared to the control group, 16.94 μg/g DW) and aromatic volatiles, while red nets increased tannins, total flavanols (17.66 μg/g DW), and aromatic volatile content. Photoselective nets have an equivalent impact on sap flow and tree water use dynamics. According to a study on apple trees, pearl nets substantially reduced sap flow and improved water usage efficiency, while red nets increased sap flow and tree water use. Blue nets provided a better-balanced trade-off between water use and fruit production, while red nets had no advantages and were considered inappropriate, since they were ineffective in directing water towards fruit growth [18].

Although the effects of photoselective nets on fruit development and quality have been the subject of much research, there are still many unanswered questions, since very few studies have explored how photoselective nets influence the phenolic composition in apples. To the best of the authors’ knowledge, no study has assessed the impact among different apple cultivars under the same edaphoclimatic conditions. Understanding how nets impact phenolic composition, including the new proposed photoselective nets, could potentially allow apple producers to improve crop quality while preserving sustainable production practices and market appeal. As a result, the purpose of this study was to assess the impact of various nets on the phenolic composition of different apple cultivars. By reviewing these interactions, we hope to determine whether specific net colors and spectrum alterations can improve the phenolic content of apples. In addition to its obvious benefits for fruit quality, this study contributes to broader agricultural objectives, such as improving post-harvest quality, advancing sustainable orchard management, and optimizing apple production under shifting environmental circumstances. The findings may lead to recommendations for the selection of photoselective nets that maximize both nutritional and economic benefits.

## 2. Results and Discussion

### 2.1. Liquid Chromatography

The detection and quantification of phenolic compounds was accomplished by using a previously validated method for 49 phenolic compounds: UHPLC-ToF-MS [19]. Seventeen phenolic compounds were quantified in at least one of the samples, and two more were identified below the limit of quantification. Identification was confirmed by comparing retention time, with a maximum relative deviation of 2.5%, and the isotope mass, with 5 ppm tolerance, when compared to the pure standards of the phenolic compounds.

The analysis of the phenolic compound composition in different portions of *Fuji aztec*, *Granny smith*, and *Gala redlum* apple cultivars grown under photoselective nets revealed distinct patterns on the phenolic composition of the fruits. The use of photoselective nets resulted in a general reduction in the production of phenolic compounds in the peels of *Gala redlum* apples, with the exceptions of gallic acid and quercetin, which showed a significant increase (*p* < 0.05). For example, quercetin levels under the lowest increase, using the IRIDIUM^®^ Yellow net, increased from 43.4 µg/g FW in the control to 88.8 µg/g FW, representing a twofold increase. Taxifolin production remained uniform in all the fruits of the same cultivar, suggesting that this compound may be less sensitive to the conditions imposed by the nets. Chlorogenic acid was the most heavily affected compound, with reductions ranging from 42 to 77%, possibly indicating that its synthesis might be highly dependent on sunlight, in addition to genetic factors. Light exposure stimulates the activity of enzymes like phenylalanine ammonia-lyase (PAL), which is involved in phenolic biosynthesis [20]. However, excessive sunlight or high temperatures can also lead to phenolic degradation. Quercetin more than doubled in quantity in the fruits produced under nets (43.44 +0.261 µg/g FW in the control group and 153.2 + 1.586 µg/g FW in the peels of the fruits produced under the gray net), while quercetin-3-β-D-glucoside only increased in the gray net, halving in the others, with both showing significant differences (*p* < 0.05). A similar level of quercetin in the peels of apple was previously reported by Gulsunoglu et al. [21]. As highlighted by Yang et al. [22], widely targeted metabolomics approaches can reveal detailed metabolic responses to environmental stimuli, such as light quality. In our study, the increased levels of quercetin observed under the gray and IRIDIUM^®^ Red nets may be linked to changes in the spectral composition of transmitted light. Taxifolin showed a small increase in the gray net but decreased in the other nets. In the peels of the *Fuji aztec* cultivars, the production of chlorogenic acid, phloridzin, and 4-o-caffeoylquinic acid significantly decreased in all the nets except the gray net (*p* < 0.05). The effect of photoselective nets on the phenolic profile of *Fuji* apples was previously studied by Bastías et al. [23], and the authors revealed that the level of quercetin-3-β-D-glucoside diminished in the fruits grown under nets. This is in line with the results obtained in the present study. In contrast, quercitrin and cyanidin-3-glucoside showed increases in the krystal net compared to the control, while rutin production increased in both the krystal and gray nets. Kaempferol-3-O-β-rutinoside showed a significant increase in production, tripling in concentration in the gray net, suggesting a promoting effect of this net on the biosynthesis of this specific compound (*p* < 0.05). The variation in phenolic compounds in the peel of the *Granny smith* cultivar was less uniform, with some nets promoting increases while others caused reductions. The IRIDIUM^®^ Yellow, IRIDIUM^®^ Red, and krystal nets increased the production of more than 50 per cent of the phenolic compounds detected, resulting in a higher total phenolic compound content than the control. Quercetin increased by between 25 and 50% in the IRIDIUM^®^ Yellow and krystal nets, while rutin and kaempferol-3-O-β-rutinoside increased threefold in the krystal net. A complete look at these findings can be found in Table 1.

**Figure 1:**
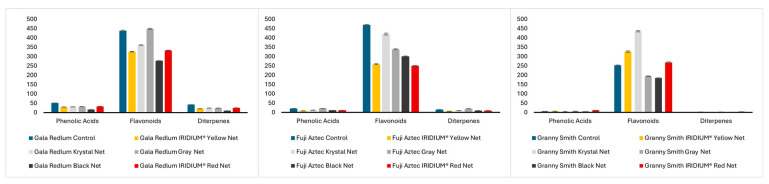
Sum of the individual phenolic compounds by class in the peels of *Gala redlum*, *Fuji aztec*, and *Granny smith* apples. Results are expressed in µg/g of fresh fruit as mean ± standard deviation, from three replicates.

As can be seen in Figure 1, flavonoids are the major component of peels when compared to other phenolic compound classes like phenolic acids and diterpenes. According to Zoratti et al. [24], light plays a crucial role in flavonoid biosynthesis, as many key enzymes in this pathway are regulated by light-responsive transcription factors. Reducing sunlight exposure through photoselective nets often leads to a decrease in flavonoid accumulation. This effect has been observed in apples [25], nectarines [26], and pears [27], where lower light availability downregulates genes involved in flavonoid synthesis, leading to reduced content. However, the response varies depending on fruit species, developmental stage, and the spectral composition of available light. The control group showed a total sum of individual flavonoids of 438.6 + 2.812 and 469.3 + 1.753 µg/g FW for the *Gala redlum* and *Fuji aztec* cultivars, respectively, but only 252.5 + 1.692 µg/g FW for the *Granny smith* cultivar. The IRIDIUM^®^ Yellow, IRIDIUM^®^ Red, and mainly the krystal net totaled a sum of flavonoids higher than the control group regarding *Granny smith* apples.

Also, in the *Fuji aztec* cultivar, the krystal net performed better than the other nets, providing fruits with similar flavonoid contents (420.2 + 5.288 µg/g FW) to the control group. However, it was the gray net that proved to produce better-quality fruits of the *Gala redlum* cultivar, providing phenolic acid, flavonoid, and diterpene contents very similar to those of the control group.

Regarding the seeds, in the *Gala redlum* cultivar, a trend similar to that of the peels was observed, but with a greater increase in phenolic compounds. Gallic acid increased slightly but not significantly (*p* ≥ 0.05) in all nets, suggesting that this compound may be less dependent on direct exposure to light. O-coumaric acid increased only in the IRIDIUM^®^ Yellow net. The phloridzin content remained stable in the IRIDIUM^®^ Yellow net but decreased by about half in the other nets. The gray and IRIDIUM^®^ Red nets promoted an increase in the production of quercetin, quercetin-3-β-D-glucoside, kaempferol-3-O-β-rutinoside, and rutin. In the seeds of the *Fuji aztec* cultivar, catechin showed a slight significant increase in all the nets except the black and IRIDIUM^®^ Red nets. O-coumaric acid increased slightly only in the gray net. The krystal and black nets favored the production of quercetin and quercetin-3-β-D-glucoside. Quercitrin, which was below the limit of quantification (1.415 µg/g FW) in the control, was detected in higher concentrations in the krystal and IRIDIUM^®^ Red nets. Kaempferol-3-O-β-rutinoside increased only in the black net, which may indicate a specific response of this network to the wavelength of light captured by the net. Contrarily to what was registered in the peels, the phenolic compounds detected in the seeds of the *Granny smith* cultivar showed higher concentrations in the control than in the nets, with the exception of the IRIDIUM^®^ Red net, where increases were observed in several compounds. Both o-coumaric acid and vanillic acid were detected below the LOQ (2.609 and 1.374 µg/g FW, respectively) in the control, but above it in the IRIDIUM^®^ Red net. Phloridzin increased in the IRIDIUM^®^ Red, crystal, and black nets. Quercetin significantly increased in all nets (*p* < 0.05), while kaempferol-3-O-β-rutinoside more than doubled in the IRIDIUM^®^ Yellow and gray nets. A broader look at the individual phenolic compound content found in the seeds of the apples studied can be seen in Table 2.

**Figure 2:**
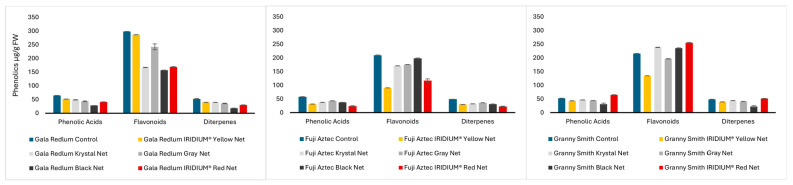
Sum of the individual phenolic compounds by class in the seeds of *Gala redlum*, *Fuji aztec*, and *Granny smith* apples. Results are expressed in µg/g of fresh fruit as mean ± standard deviation, from three replicates.

Figure 2 reveals that the seeds had lower amounts of flavonoids, but phenolic acids and diterpenes seemed more abundant in seeds compared to peels. Again, in this portion the *Gala redlum* and *Fuji aztec* cultivars were revealed to have more total flavonoids and phenolic acids in the control group than in the groups cultivated under nets. The same is not true for the *Granny smith* cultivar, since the gray and black nets produced fruits with higher flavonoid contents and the IRIDIUM^®^ Red net produced fruits with both higher flavonoid and phenolic acid contents. The diterpene content was similar in those two groups.

Remarkably, the seeds of the fruits produced under the IRIDIUM^®^ Yellow net had phloridzin contents of 255.6 + 0.123 µg/g FW, which are very similar to those displayed by the Pêro de Borbela cultivar (318.7 µg/g) previously reported as a standout in a study contemplating Portuguese traditional cultivars [19] but much lower than all the seeds reported in the work of Xu et al. [28] (2405–8644 µg/g), although in dry weight.

In the pulps of the three varieties, the concentrations of the phenolic compounds remained relatively constant.

The results suggest that photoselective nets have a different impact on the phenolic composition of the different parts of an apple. The reduction in chlorogenic acid content was one of the most consistent effects, suggesting a direct dependence of this compound on light conditions. On the other hand, some nets seemed to promote quercetin and other flavonoids, especially the gray and IRIDIUM^®^ Red ones.

### 2.2. Antioxidant Capacity Assays

**Figure 3:**
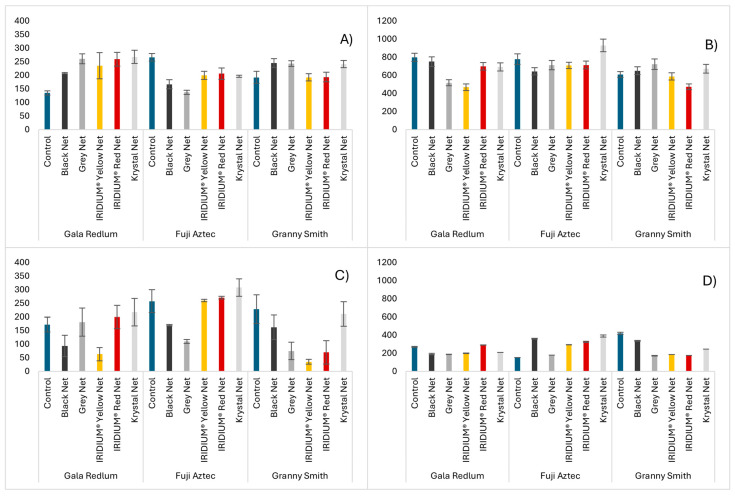
Antioxidant capacity of apples from the different cultivars and nets evaluated using the β-Carotene bleaching assay ((**A**)—peels, (**C**)—seeds) in AAC and DPPH radical scavenging assay ((**B**)—peels, (**D**)—seeds) in µg TE/g FW. Results are expressed as mean ± standard deviation (n = 3).

β-carotene bleaching is a very commonly used assay to evaluate the antioxidant activity of a determined substance or mixture. The term “bleaching” refers to the loss of color by the mixture, which is caused by the disruption of the β-carotene molecule by the action of free radicals produced by linoleic acid oxidation. When phenolic compounds are present in the mixture, they retard the bleaching by capturing the free radicals that would attack the β-carotene molecule [29]. Apple extracts revealed that in the peel portion (Figure 3A), the control group showcased a lower AAC than the fruits produced under nets in the *Gala redlum* and *Granny smith* cultivars. In the *Fuji aztec* cultivar, however, it was the control group that produced fruits with higher antioxidant power. In the seed portion (Figure 3C), the krystal net showed a higher ability to produce fruits with a greater AAC than the control group across all cultivars. The IRIDIUM^®^ Red net is also noteworthy for achieving this in the *Gala redlum* and *Fuji aztec* cultivars.

There are many commonly used methods to determine the antioxidant capacity of fruits [30,31], but the DPPH radical scavenging assay is widely considered to be the most popular of them [32]. As described before, the peels revealed higher antioxidant capacity than the seeds, but as the total content of individual phenolic acids and diterpenes found on the seeds was higher than the amount found on the peels, it is intriguing to consider whether flavonoids could be responsible for most of the antioxidant activity recorded in these portions. Regarding the effect of the nets on the apple peels (Figure 3B), the results were as expected for the *Gala redlum* and *Fuji aztec* cultivars, but in the *Granny smith* cultivar, three of the nets actually increased the antioxidant capacity of the fruits. In the seeds (Figure 3D), the contrary happened, and the *Granny smith* cultivar behaved as expected, while all the *Fuji aztec* grown under nets demonstrated increased antioxidant activity. Abbasnia Zare et al. [33] also described plants having increased antioxidant capacity when cultivated under nets, although these were ornamental plant species, but the same authors reported a lower capacity for two foliage plant species produced under yellow and red nets [34].

**Figure 4:**
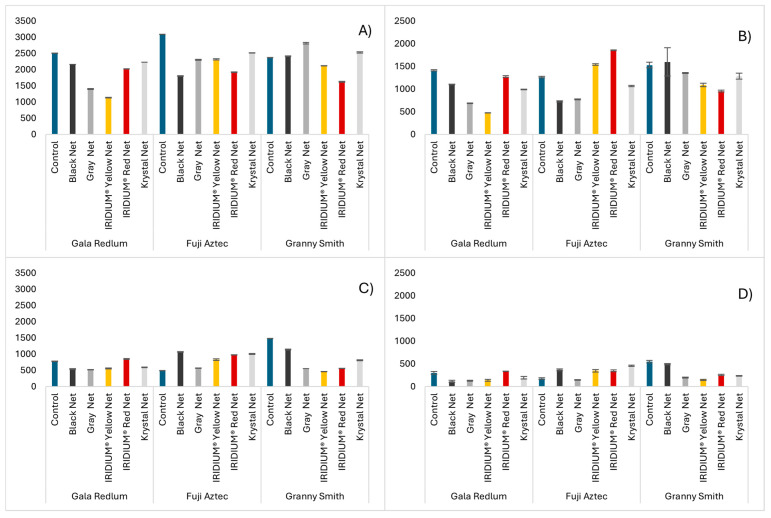
Total phenolic content of apples from the different cultivars and nets evaluated ((**A**)—peels, (**C**)—seeds) in µg GAE/g FW and total flavonoid content of the same samples ((**B**)—peels, (**D**)—seeds) in µg EE/g FW. Results are expressed as mean ± standard deviation (n = 3).

Similarly to what was described regarding the total sums of individual phenolic compounds, the total phenolic content of the peels of the *Gala redlum* and *Fuji aztec* cultivars was higher in the control groups than in the fruits grown under nets, and again, the *Granny smith* cultivar showcased peels with a higher total phenolic content for fruits grown under the gray net (Figure 4A). In the seeds (Figure 4C), however, in addition to having much lower contents than the peels, the *Gala redlum* IRIDIUM^®^ Red nets produced fruits with higher TPC than the control group. Together with all the nets having produced fruits richer in phenolic compounds than the control group in the *Fuji aztec* cultivar and less rich in the *Granny smith* cultivar, this represents the opposite of what was found when analyzing individual phenolic compounds. This could mean that there are additional phenolic compounds present in the seeds of these fruits that are not being determined by the UHPLC-Tof-MS method.

The assessment of the total flavonoid content (Figure 4B,D) showed that the control group of *Gala redlum* apple peels possessed almost three times the flavonoid content that the fruits from the IRIDIUM^®^ Yellow net exhibited and nearly twice the flavonoid content of those from the gray net, contrasting with the individual phenolic determination, where the flavonoid levels in the control group were comparable to those from the IRIDIUM^®^ Yellow net and lower than those from the gray net. This finding aligns with our previous hypothesis and further suggests that certain compounds that were not detected by the chromatographic method may belong to the flavonoid subclass.

## 3. Materials and Methods

### 3.1. Reagents

Aluminum chloride, β-Carotene, chloroform, DPPH (2,2-diphenyl-1-picrylhydrazyl), Folin–Cioucalteu reagent, linoleic acid, sodium carbonate, sodium hydroxide, sodium nitrite, Trolox ((±)-6-hydroxy-2,5,7,8-tetramethylchromane-2-carboxylic acid), and Tween^®^ 40 and the phenolic compound standards ((-)-epigallocatechin, (−)-epigallocatechin gallate, (−)-gallocatechin, (−)-gallocatechin gallate, 4-hydroxybenzoic acid, 4-o-caffeoylquinic acid, 5,7-dimethoxyluteolinidin chloride, apigenin, apigeninidin chloride, caffeic acid, carnosic acid, catechin, chlorogenic acid, chrysin, cyanidin-3-glucoside, cynarin, ellagic acid, epicatechin, epicatechin gallate, eriocitrin, eriodictyol, gallic acid, genistein, genistin, hesperidin, isorhamnetin-3-o-glucoside, kaempferol-3-o-b-rutinoside, luteolin, luteolin-7-o-glucoside, luteolinidin chloride, myricetin, naringenin, narirutin, neochlorogenic acid, o-coumaric acid, p-coumaric acid, phloridzin, pinocembrin, quercetin, quercetin-3-b-d-glucoside, quercitrin, rosmarinic acid, rutin, sakuranetin, sinapic acid, sinensetin, syringic acid, tangeretin, taxifolin; purity ≥ 95.0%) were all purchased from Sigma Aldrich. Solvents ethanol and methanol were purchased from Honeywell. Ultra-pure water was obtained from a Milli-Q plus system from Millipore.

### 3.2. Apple Cultivars and Photoselective Nets

Three apple cultivars from Alcobaça (Portugal) were chosen for this study. The selected cultivars were *Gala redlum*, *Granny smith*, and *Fuji aztec*, chosen because of their global importance and because they are among the most widely produced in Portugal. In addition to the control group (no nets), 5 nets were chosen to be installed over the apple crops—black (SF ≈ 22.4%), IRIDIUM^®^ Red (SF ≈ 20.6%), IRIDIUM^®^ Yellow (SF ≈ 14.7%), krystal (SF ≈ 9.6%) and gray (SF ≈ 15.7%). Shading factors (SFs) were measured in the field at solar noon, with a spectroradiometer (Apogee, mod. SS110, Logan, UT, USA), showing small differences to the nominal values presented by the providers (black, gray and krystal HDPE nets, 2.80 × 8.50 mm mesh, Type Austria^®^, Artes Politécnica, Italy; IRIDIUM^®^ Yellow and Red HDPE nets, 2.40 × 4.80 mm mesh, Agrintech, Pezza Grande, Italy). This orchard was established in 2018, with a tree density of 3175 trees per hectare (3.50 × 0.90 m), at an experimental station (INIAV, IP) located in the Alcobaça/Portugal region (Lat. 39,549; Long. −8.959) and covered in 2020, with an experimental design consisting of at least three consecutive rows with the same type of net, ensuring that fruits were harvested from the center row.

### 3.3. Preparation of the Samples

Ten to twelve apples of each cultivar, with the most representative diameter obtained after calibration (65–70 mm for *Gala redlum*; 70–75 mm for *Granny smith* and *Fuji aztec*), were selected for analysis. Apples were harvested in the state of ripeness best suited for the Portuguese market needs. Fifty apples from the most representative diameter of each cultivar were measured for (i) fruit firmness (FF), using a Penefel^®^ penetrometer (Setop Giraud Technologie, Cavaillon, France), with an 11 mm diameter probe, according to the manufacturer’s protocol, with results expressed in kg/cm^2^; (ii) soluble solid content (SSC), using a digital refractometer (HI96801, Hanna Instruments, Woonsocket, RI, USA), with results expressed in °Brix; and (iii) starch index (SI), based on the rate of starch-to-sugar conversion in the flesh, using the iodine test and rated on a 1 to 10 scale according to the CTIFL starch index chart where 1 indicates full starch presence and 10 represents complete starch hydrolysis. The results obtained at harvest were (*Gala redlum*: FF 6.36 ± 0.24; SSC 12.96 ± 0.44; SI 7.93 ± 0.47; *Granny smith*: FF 6.84 ± 0.20; SSC 11.22 ± 0.30; SI 6.45 ± 0.47; *Fuji Aztec*: FF 6.64 ± 0.20; SSC 14.28 ± 0.41; SI 9.01 ± 0.12). A summary of these charachteristics can be found in Table 3.

Each fruit was separated into three distinct fractions: peel, seeds, and mesocarp. These fractions were individually homogenized using a standard homogenizer (Ultra-Turrax^®^ T25, Janke and Kunkel IKA, Stavfen, Germany). For the antioxidant capacity assays, extracts were obtained by combining two grams of the homogenized sample with 20 mL of 95% ethanol (*v*/*v*). The mixture was processed using an Ultra-Turrax homogenizer for three minutes, followed by centrifugation at 2250× *g* for 10 min at 20 °C. The supernatant was collected, and the residual phase was discarded. For the Ultra-High-Performance Liquid Chromatography phenolic compound analysis, two grams of the homogenized sample was mixed with 10 mL of a solvent mixture (MeOH:H_2_O:formic acid, 49.95:49.95:0.10 *v*/*v*/*v*) in a 15 mL Falcon tube. The mixture was subjected to sonication at room temperature for 10 min and then agitated for 15 min using a horizontal shaker. After centrifugation at 2250× *g* for 10 min at 20 °C, the supernatant was transferred to a separate Falcon tube. The extraction was repeated with an additional 10 mL of the same solvent, and both extracts were combined to obtain the final sample.

### 3.4. Chromatography Instrumentation

Detection of phenolic compounds was performed utilizing an Ultra-High-Performance Liquid Chromatography–Time of Flight–Mass Spectrometry (UHPLC-ToF-MS) system comprising a Nexera X2 Shimadzu UHPLC, which included a solvent degasser, a binary pump, an autosampler, an automatic injector, and an oven for the column, as well as a 5600+ ToF-MS detector (SCIEX, Foster City, CA, USA) equipped with a Turbo Ion Spray electrospray ionization source, in positive mode (ESI+). The column used was an Acquity UPLC BEH C18 (2.1 mm × 100 mm, 1.7 μm) in gradient mode, following the subsequent gradient 0–0.5 min at 90% (Solvent A), 0.5–8 min from 90% to 20% (Solvent A), and, until the end of the run, 20% (Solvent A), totaling a run time of 8.1 min. The mobile phase was an aqueous solution of 0.1% formic acid (Solvent A) and acetonitrile with 0.1% formic acid (Solvent B). Acquisition was performed in full-scan mode, with a range between 100 and 750 Da. The selected ion source voltage was 5500 V; the source temperature was 575 °C; the curtain gas was 30 psi; Gas 1 and Gas 2 were 55 psi; and the declustering potential (DP) was 100 V. Two types of software were used for data processing and phenolic compound identification —PeakView™ 2.2 and MultiQuant™ 3.0 software (SCIEX, Foster City, CA, USA). PeakView™ 2.2 software automatically presented the isotope match. Two parameters and their accompanying equations (Equations (1) and (2)) were employed for phenolic compound identification and were based on the CIR 808/2021 requirements: (1) maximum retention time deviation (∆RT) of 0.1 min (Equation (1)); and (2) exact mass deviation (m) with a tolerance of 5 ppm (Equation (2)).

(1) $$\mathrm{RT}=\left(\frac{\mathrm{RT}\;\mathrm{spiked}\;\mathrm{samples}\;-\;\mathrm{RT}\;\mathrm{standard}}{\mathrm{RT}\;\mathrm{standard}}\right)\times 100$$

(2) $$\Delta m\;\left(\mathrm{ppm}\right)=\left(\frac{\mathrm{Exact}\;\mathrm{mass}\;-\;\mathrm{Detected}\;\mathrm{mass}}{\mathrm{Exact}\;\mathrm{mass}}\right)\times 10^{6}$$

where *RT* is the retention time and ∆m is the exact mass deviation.

### 3.5. Antioxidant Capacity Assays

1. β-Carotene bleaching assay

The assessment of the antioxidant activity was performed by a slight modification of Miller’s method [35]. A β-carotene/linoleic acid emulsion was prepared by mixing 1 mL of β-carotene in chloroform solution (0.2 mg/mL) with 20 mg of linoleic acid and 200 mg of Tween^®^ 40. The chloroform was evaporated in a rotary evaporator, and 100 mL of ultrapure water was added. The solution was vigorously shaken until the emulsion was formed. An aliquot of 200 µL of the sample and 5 mL of the β-carotene emulsion were mixed and maintained at 55 °C for two hours in a water bath, and the absorbance was read in a spectrophotometer at 470 nm. The antioxidant activity coefficient (AAC) was calculated following Equation (3):

(3) $$\mathrm{AAC}=\left(\frac{Abs\;sample-A120\;blank}{A0\;blank-A120\;blank}\right)\times 100$$

where A0 blank is the absorbance of the blank solution at t = 0 min, A120 blank is the absorbance of the blank at t = 120 min, and Abs sample is the absorbance of the sample after t = 120 min. All experiments were conducted using triplicates.

2. DPPH radical scavenging assay

To test the phenolic compounds’ ability to scavenge free radicals and, consequently, assess the extract’s antioxidant potential, the method developed by Martins et al. [36] was utilized. In short, a 15 mL Falcon tube was filled with 50 µL of sample and 2 mL of the DPPH radical solution (14.2 µg/mL). The tube was then left in the dark for 30 min, and the absorbance was measured at 515 nm. Trolox was used as a standard, and the Trolox equivalents (TEs) were measured in µg per gram of fruit (µg TE/g). All experiments were conducted using triplicates.

3. Total phenolic content assay

The method developed by Erkan et al. [37] was used to determine the fruit extracts’ total phenolic content. This was accomplished by mixing an aliquot of 1 mL of the sample with 7.5 mL of 10% *v*/*v* Folin–Cioucalteu reagent, letting it sit for 5 min, and then adding 7.5 mL of Na_2_CO_3_ (60 mg/mL). After 120 min, the absorbance at 725 nm was measured. Gallic acid was used as a standard. Results were expressed as µg gallic acid equivalents (GAEs) per gram of fruit. Every experiment was carried out in triplicate.

4. Total flavonoid content assay

The total flavonoid content was determined using a method described by Barbosa et al. [38]. In summary, 0.3 mL of sodium nitrite (50 mg/mL) and 4 mL of ultrapure water were mixed with 1 mL of the sample. After five minutes, 100 mg/mL of aluminum chloride was added to the mixture. Six minutes later, 40 mg/mL sodium hydroxide and 2.1 mL of ultrapure water were added. The absorbance was read at 510 nm, and epicatechin was used as the standard. The results were presented as µg epicatechin equivalents (EEs)/g of fruit. Every experiment was carried out in triplicate.

### 3.6. Statistical Analysis

A two-way analysis of variance (ANOVA) was used to assess how the cultivar, net, and portion of the fruit affected the samples’ phenolic profile and antioxidant properties. Significance was determined at *p* < 0.05. All data analyses were carried out using RStudio 2024.04.2 and Microsoft Excel 365 with Analysis ToolPak installed.

## 4. Conclusions

To the best of our knowledge, this study is the first to investigate the effects of different photoselective nets on various apple cultivars grown under the same edaphoclimatic conditions, making it a novel contribution to the field. This study illustrates the strong effect of photoselective nets on the phenolic composition and antioxidant activities of apples. The results reveal that different net colors have distinct effects on the accumulation of phenolic compounds, with certain nets increasing flavonoid production and others decreasing the amount of important phenolic acids. In particular, the gray and IRIDIUM^®^ Red nets enhanced the production of quercetin and its derivatives, whereas chlorogenic acid showed a general decline under net-covered conditions, reinforcing its dependence on direct sunlight exposure. Gray nets typically allow more blue and UV-A light to reach the plant canopy, which are known to activate photoreceptors such as UVR8 and cryptochromes. This activation can trigger responses involving regulators like MYB12, MYB111, and HY5, which promote the biosynthesis of flavonols including quercetin. While our experimental design did not include spectral measurements, these findings are consistent with known light–phenolic interactions.

The findings also show cultivar-specific reactions, with *Gala redlum* apples demonstrating the most severe decreases in phenolic compounds under nets, while *Granny smith* apples showed an increase in overall phenolic content under specified net conditions. The antioxidant assays support the chromatographic findings, demonstrating that photoselective nets influence the antioxidant potential of apples in a cultivar-dependent manner.

These findings are of utmost importance for orchard management procedures, potentially allowing producers to improve fruit quality and bioactive ingredient retention by strategically selecting netting materials. Future research should investigate the long-term physiological and molecular mechanisms underlying these cultivar-specific responses to photoselective nets, particularly the regulation of flavonoid biosynthesis pathways, as well as their implications for post-harvest quality and consumer health. Furthermore, extending this research across multiple growing seasons and different geographical locations would provide deeper insight into the long-term effects of netting on apple quality. Investigating other bioactive compounds beyond phenolics, such as vitamins and carotenoids, could further enhance our understanding of the impact of light manipulation on apple nutritional value. Finally, assessing the sensory attributes and post-harvest stability of apples grown under photoselective nets would be valuable for determining their commercial viability.

## Figures and Tables

**Table 1:** Individual phenolic compound contents, in µg per gram of fresh weight (µg/g FW), in the peels of 3 apple cultivars produced under different nets, including IRIDIUM^®^ Yellow, krystal, gray, black, and IRIDIUM^®^ Red nets, determined by UHPLC-ToF-MS. Results (µg/g FW) are expressed as mean ± standard deviation (SD), from three replicates. LOQ–limit of quantification. n.d.—not detected. Superscript letters (a, b, c, d) in each row denote statistically significant differences (*p* < 0.05) between treatments for the same compound and cultivar.

Phenolic Compound	*Gala redlum*						*Fuji aztec*						*Granny smith*					
	Control	Yellow	Krystal	Gray	Black	Red	Control	Yellow	Krystal	Gray	Black	Red	Control	Yellow	Krystal	Gray	Black	Red
4-Hydroxybenzoic Acid	4.957 ^a^ + 0.005	3.717 ^bc^ + 0.008	3.205 ^c^ + 0.023	3.538 ^bc^ + 0.014	2.072 ^d^ + 0.006	3.844 ^b^ + 0.060	1.350 ^a^ + 0.029	n.d.	n.d.	<LOQ	n.d.	n.d.	1.481 ^a^ + 0.002	1.770 ^a^ + 0.026	0.869 ^b^ + 0.030	1.572 ^a^ + 0.001	0.922 ^b^ + 0.016	1.656 ^a^ + 0.044
4-O-Caffeoylquinic Acid	42.57 ^a^ + 0.051	21.32 ^c^ + 0.032	24.01 ^b^ + 0.044	24.24 ^b^ + 0.051	9.914 ^d^ + 0.055	24.93 ^b^ + 0.141	15.46 ^b^ + 0.448	7.528 ^c^ + 0.009	10.87 ^c^ + 0.009	20.13 ^a^ + 0.041	10.01 ^c^ + 0.020	9.979 ^c^ + 0.033	1.548 ^b^ + 0.012	2.575 ^ab^ + 0.000	1.492 ^b^ + 0.052	2.036 ^b^ + 0.001	<LOQ	3.979 ^a^ + 0.160
Carnosic Acid	n.d.	n.d.	n.d.	<LOQ	<LOQ	<LOQ	n.d.	<LOQ	<LOQ	n.d.	<LOQ	<LOQ	n.d.	n.d.	n.d.	n.d.	n.d.	n.d.
Catechin	6.086 ^a^ + 0.024	4.716 ^bc^ + 0.006	3.874 ^d^ + 0.014	4.255 ^cd^ + 0.019	1.870 ^e^ + 0.004	5.123 ^b^ + 0.048	1.581 ^ab^ + 0.042	<LOQ	1.243 ^bc^ + 0.001	1.737 ^a^ + 0.016	1.363 ^ab^ + 0.001	0.934 ^c^ + 0.002	3.692 ^ab^ + 0.031	4.634 ^a^ + 0.003	2.369 ^b^ + 0.065	4.295 ^a^ + 0.013	2.357 ^b^ + 0.007	4.688 ^a^ + 0.221
Chlorogenic Acid	42.66 ^a^ + 0.051	21.41 ^c^ + 0.030	23.84 ^b^ + 0.043	24.08 ^b^ + 0.051	9.987 ^d^ + 0.055	25.01 ^b^ + 0.144	15.55 ^b^ + 0.444	7.546 ^c^ + 0.003	10.93 ^c^ + 0.009	20.20 ^a^ + 0.042	10.07 ^c^ + 0.020	10.03 ^c^ + 0.032	1.577 ^b^ + 0.010	2.590 ^ab^ + 0.002	1.537 ^b^ + 0.053	2.066 ^b^ + 0.003	<LOQ	3.966 ^a^ + 0.156
Cyanidin-3-Glucoside	69.59 ^a^ + 0.459	55.86 ^b^ + 0.057	44.70 ^c^ + 0.339	30.29 ^e^ + 0.057	27.65 ^e^ + 0.337	38.98 ^d^ + 0.195	44.95 ^b^ + 0.205	26.71 ^d^ + 0.106	55.03 ^a^ + 0.416	25.21 ^d^ + 0.186	36.37 ^c^ + 0.439	19.60 ^e^ + 0.107	n.d.	n.d.	n.d.	n.d.	n.d.	n.d.
Epicatechin	49.72 ^a^ + 0.042	37.43 ^bc^ + 0.321	33.47 ^d^ + 0.214	34.87 ^cd^ + 0.041	20.84 ^e^ + 0.036	39.52 ^b^ + 0.211	31.26 ^a^ + 0.456	14.50 ^cd^ + 0.106	18.11 ^bc^ + 0.096	18.70 ^b^ + 0.033	19.29 ^b^ + 0.011	13.82 ^d^ + 0.180	22.12 ^bc^ + 0.283	22.93 ^b^ + 0.097	16.32 ^d^ + 0.117	19.46 ^c^ + 0.080	13.82 ^d^ + 0.018	30.75 ^a^ + 0.173
Gallic Acid	3.858 ^d^ + 0.006	4.249 ^ab^ + 0.011	4.018 ^cd^ + 0.003	4.148 ^bc^ + 0.010	4.352 ^a^ + 0.002	3.900 ^d^ + 0.011	4.356 ^a^ + 0.062	1.645 ^b^ + 0.003	1.340 ^b^ + 0.003	1.482 ^b^ + 0.028	1.596 ^b^ + 0.026	1.887 ^b^ + 0.004	2.808 ^c^ + 0.021	3.034 ^c^ + 0.004	2.682 ^c^ + 0.014	2.814 ^c^ + 0.065	4.190 ^b^ + 0.031	6.160 ^a^ + 0.002
Isorhamnetin-3-O-Glucoside	0.884 ^a^ + 0.001	<LOQ	<LOQ	0.791 ^b^ + 0.004	<LOQ	<LOQ	<LOQ	n.d.	<LOQ	n.d.	n.d.	n.d.	1.149 ^a^ + 0.005	1.009 ^ab^ + 0.012	0.804 ^c^ + 0.003	0.913 ^bc^ + 0.011	0.775 ^c^ + 0.014	1.145 ^a^ + 0.017
Kaempferol-3-O-β-Rutinoside	22.23 ^a^ + 0.147	6.353 ^e^ + 0.022	8.213 ^d^ + 0.038	18.43 ^b^ + 0.027	12.89 ^c^ + 0.005	9.298 ^d^ + 0.055	17.02 ^b^ + 0.049	4.941 ^d^ + 0.011	13.53 ^bc^ + 0.748	51.47 ^a^ + 0.278	7.150 ^d^ + 0.042	10.14 ^cd^ + 0.050	27.53 ^bc^ + 0.155	34.57 ^b^ + 1.286	86.97 ^a^ + 1.402	8.657 ^d^ + 0.104	14.50 ^cd^ + 0.267	13.65 ^cd^ + 0.590
o-Coumaric Acid	n.d.	<LOQ	n.d.	n.d.	n.d.	n.d.	n.d.	n.d.	n.d.	n.d.	n.d.	n.d.	n.d.	n.d.	n.d.	n.d.	n.d.	n.d.
*p*-Coumaric Acid	n.d.	<LOQ	n.d.	n.d.	n.d.	n.d.	n.d.	n.d.	n.d.	n.d.	n.d.	n.d.	n.d.	n.d.	n.d.	n.d.	n.d.	n.d.
Phloridzin	21.62 ^a^ + 0.125	12.65 ^d^ + 0.057	15.60 ^c^ + 0.020	16.15 ^c^ + 0.039	6.760 ^e^ + 0.005	19.44 ^b^ + 0.026	25.21 ^ab^ + 0.130	19.76 ^c^ + 0.030	24.32 ^b^ + 0.037	26.91 ^a^ + 0.001	21.55 ^c^ + 0.155	17.94 ^d^ + 0.090	6.242 ^cd^ + 0.044	6.037 ^cd^ + 0.073	8.724 ^a^ + 0.002	6.790 ^bc^ + 0.038	5.406 ^d^ + 0.046	7.296 ^b^ + 0.057
Quercetin	43.44 ^d^ + 0.261	88.83 ^c^ + 1.200	118.7 ^b^ + 0.263	153.2 ^a^ + 1.586	109.5 ^b^ + 0.285	105.8 ^b^ + 1.128	142.9 ^a^ + 0.162	92.28 ^b^ + 1.736	129.4 ^a^ + 1.800	81.41 ^b^ + 0.213	93.75 ^b^ + 0.122	84.15 ^b^ + 0.317	90.70 ^c^ + 0.388	125.2 ^a^ + 0.728	121.0 ^ab^ + 0.000	82.12 ^cd^ + 1.120	74.85 ^d^ + 0.140	109.0 ^b^ + 0.689
Quercetin-3-β-D-Glucoside	141.5 ^a^ + 0.684	63.11 ^d^ + 0.101	88.10 ^b^ + 0.080	147.4 ^a^ + 0.402	70.26 ^c^ + 0.044	72.34 ^c^ + 0.143	140.3 ^a^ + 0.438	69.72 ^c^ + 0.063	104.9 ^b^ + 1.949	86.51 ^c^ + 0.804	78.22 ^c^ + 0.147	73.61 ^c^ + 0.454	80.95 ^cd^ + 0.678	107.1 ^b^ + 1.205	144.6 ^a^ + 1.520	63.82 ^de^ + 0.508	60.95 ^e^ + 0.197	87.23 ^c^ + 1.099
Quercitrin	68.30 ^a^ + 0.995	55.08 ^b^ + 0.006	43.34 ^c^ + 0.043	29.09 ^d^ + 0.062	23.32 ^d^ + 0.037	37.79 ^c^ + 0.147	45.13 ^b^ + 0.223	25.77 ^d^ + 0.086	51.28 ^a^ + 0.147	24.22 ^d^ + 0.361	33.60 ^c^ + 0.245	18.14 ^e^ + 0.034	n.d.	n.d.	n.d.	n.d.	n.d.	n.d.
Rutin	13.72 ^a^ + 0.063	2.234 ^d^ + 0.006	3.586 ^c^ + 0.012	11.90 ^b^ + 0.056	3.077 ^c^ + 0.007	3.518 ^c^ + 0.006	18.96 ^a^ + 0.028	4.784 ^c^ + 0.005	20.63 ^a^ + 0.089	22.37 ^a^ + 0.123	8.264 ^bc^ + 0.008	10.30 ^b^ + 0.169	18.84 ^c^ + 0.103	24.88 ^b^ + 0.275	52.00 ^a^ + 0.304	8.103 ^e^ + 0.080	12.03 ^d^ + 0.042	12.69 ^d^ + 0.074
Taxifolin	1.455 ^b^ + 0.006	<LOQ	1.134 ^c^ + 0.014	1.746 ^a^ + 0.001	1.372 ^b^ + 0.006	1.106 ^c^ + 0.000	1.915 ^a^ + 0.017	<LOQ	1.647 ^b^ + 0.003	<LOQ	1.500 ^b^ + 0.002	1.454 ^b^ + 0.015	1.338 ^c^ + 0.000	<LOQ	1.935 ^a^ + 0.005	<LOQ	<LOQ	1.629 ^b^ + 0.002
Vanilic Acid	n.d.	n.d.	n.d.	n.d.	n.d.	n.d.	n.d.	n.d.	n.d.	n.d.	n.d.	n.d.	n.d.	n.d.	n.d.	n.d.	n.d.	n.d.

**Table 2:** Individual phenolic compound content, in µg per gram of fresh weight (µg/g FW), in the seeds of 3 apple cultivars produced under different nets, including IRIDIUM^®^ Yellow, krystal, gray, black, and IRIDIUM^®^ Red nets, determined by UHPLC-ToF-MS. Results (µg/g FW) are expressed as mean ± standard deviation (SD), from three replicates. LOQ—limit of quantification. n.d.—not detected. Superscript letters (a, b, c, d) in each row denote statistically significant differences (*p* < 0.05) between treatments for the same compound and cultivar.

Phenolic Compound	*Gala redlum*						*Fuji aztec*						*Granny smith*					
	Control	Yellow	Krystal	Gray	Black	Red	Control	Yellow	Krystal	Gray	Black	Red	Control	Yellow	Krystal	Gray	Black	Red
4-Hydroxybenzoic Acid	<LOQ	<LOQ	<LOQ	<LOQ	<LOQ	<LOQ	n.d.	n.d.	n.d.	<LOQ	n.d.	n.d.	1.390 ^b^ + 0.009	1.170 ^c^ + 0.003	<LOQ	1.020 ^d^ + 0.007	0.921 ^d^ + 0.007	2.080 ^a^ + 0.011
4-O-Caffeoylquinic Acid	52.44 ^a^ + 0.557	39.76 ^b^ + 0.181	39.72 ^b^ + 0.446	35.42 ^b^ + 1.279	18.44 ^c^ + 0.062	30.35 ^b^ + 0.266	49.35 ^a^ + 0.079	30.29 ^bc^ + 0.080	32.09 ^bc^ + 0.217	36.36 ^b^ + 0.020	31.16 ^bc^ + 0.730	22.51 ^c^ + 1.075	48.66 ^a^ + 0.128	39.39 ^ab^ + 0.010	44.08 ^ab^ + 0.036	41.14 ^ab^ + 0.079	22.48 ^b^ + 2.932	52.29 ^a^ + 0.205
Carnosic Acid	n.d.	n.d.	n.d.	n.d.	n.d.	n.d.	n.d.	n.d.	n.d.	n.d.	n.d.	n.d.	n.d.	n.d.	n.d.	n.d.	n.d.	n.d.
Catechin	<LOQ	<LOQ	<LOQ	<LOQ	<LOQ	<LOQ	0.845 ^c^ + 0.001	0.868 ^c^ + 0.008	1.040 ^b^ + 0.005	1.270 ^a^ + 0.004	<LOQ	<LOQ	3.900 ^b^ + 0.027	3.570 ^c^ + 0.008	1.640 ^e^ + 0.003	3.080 ^d^ + 0.013	1.500 ^e^ + 0.011	4.640 ^a^ + 0.010
Chlorogenic Acid	52.52 ^a^ + 0.559	39.84 ^b^ + 0.181	39.73 ^b^ + 0.458	35.52 ^b^ + 1.281	18.51 ^c^ + 0.063	30.45 ^b^ + 0.267	49.46 ^a^ + 0.081	30.35 ^bc^ + 0.118	32.17 ^bc^ + 0.218	36.43 ^b^ + 0.020	31.23 ^bc^ + 0.732	22.57 ^c^ + 1.079	48.61 ^a^ + 0.156	39.46 ^ab^ + 0.011	43.87 ^ab^ + 0.036	41.23 ^ab^ + 0.081	22.54 ^b^ + 2.936	52.03 ^a^ + 0.206
Cyanidin-3-Glucoside	n.d.	n.d.	n.d.	n.d.	n.d.	n.d.	<LOQ	n.d.	4.589 ^a^ + 0.002	<LOQ	n.d.	n.d.	n.d.	n.d.	n.d.	n.d.	n.d.	n.d.
Epicatechin	6.153 ^a^ + 0.101	4.578 ^bc^ + 0.011	5.311 ^ab^ + 0.020	2.583 ^d^ + 0.109	1.775 ^d^ + 0.014	3.974 ^c^ + 0.034	7.917 ^a^ + 0.082	5.319 ^b^ + 0.022	6.293 ^ab^ + 0.007	7.593 ^ab^ + 0.011	5.683 ^ab^ + 0.043	3.953 ^b^ + 0.298	15.57 ^b^ + 0.065	15.47 ^b^ + 0.019	9.839 ^d^ + 0.002	12.02 ^c^ + 0.052	10.19 ^d^ + 0.001	19.50 ^a^ + 0.040
Gallic Acid	3.651 ^ab^ + 0.003	3.820 ^ab^ + 0.000	4.106 ^ab^ + 0.000	3.064 ^b^ + 0.088	3.862 ^ab^ + 0.001	4.854 ^a^ + 0.170	2.402 ^a^ + 0.106	1.150 ^a^ + 0.008	1.214 ^a^ + 0.000	<LOQ	1.178 ^a^ + 0.026	1.454 ^a^ + 0.124	2.540 ^c^ + 0.033	2.908 ^c^ + 0.023	2.915 ^c^ + 0.031	2.241 ^c^ + 0.017	6.897 ^a^ + 0.098	5.524 ^b^ + 0.083
Isorhamnetin-3-O-Glucoside	<LOQ	<LOQ	<LOQ	<LOQ	<LOQ	<LOQ	n.d.	n.d.	n.d.	n.d.	n.d.	<LOQ	<LOQ	<LOQ	0.787 ^a^ + 0.005	0.774 ^a^ + 0.006	0.852 ^a^ + 0.005	0.753 ^a^ + 0.007
Kaempferol-3-O-β-Rutinoside	8.084 ^bc^ + 0.009	8.428 ^bc^ + 0.109	5.120 ^c^ + 0.006	16.87 ^ab^ + 1.410	6.594 ^bc^ + 0.092	23.24 ^a^ + 0.162	20.63 ^a^ + 0.170	11.21 ^b^ + 0.130	7.473 ^bc^ + 0.035	10.23 ^b^ + 0.025	22.01 ^a^ + 0.073	5.536 ^c^ + 0.487	14.44 ^c^ + 0.101	26.38 ^b^ + 0.009	9.881 ^e^ + 0.089	33.17 ^a^ + 0.133	12.15 ^d^ + 0.050	15.94 ^c^ + 0.202
o-Coumaric Acid	6.839 ^ab^ + 0.061	8.174 ^a^ + 0.031	3.072 ^c^ + 0.009	3.429 ^c^ + 0.161	5.912 ^b^ + 0.033	5.638 ^b^ + 0.026	6.275 ^ab^ + 0.164	<LOQ	4.367 ^c^ + 0.005	6.806 ^a^ + 7.504	4.955 ^bc^ + 0.004	<LOQ	<LOQ	<LOQ	<LOQ	<LOQ	<LOQ	3.841 ^a^ + 0.074
*p*-Coumaric Acid	n.d.	n.d.	n.d.	n.d.	n.d.	n.d.	n.d.	n.d.	n.d.	n.d.	n.d.	n.d.	<LOQ	<LOQ	<LOQ	<LOQ	<LOQ	<LOQ
Phloridzin	259.9 ^a^ + 0.468	255.6 ^a^ + 0.123	142.2 ^bc^ + 0.782	181.0 ^b^ + 5.928	127.3 ^c^ + 0.315	106.3 ^c^ + 0.384	146.8 ^a^ + 0.831	60.43 ^c^ + 0.314	100.6 ^b^ + 0.014	135.6 ^a^ + 0.062	131.0 ^a^ + 1.245	80.71 ^bc^ + 3.326	140.1 ^d^ + 0.076	61.65 ^f^ + 0.038	184.7 ^a^ + 0.502	105.3 ^e^ + 0.428	175.3 ^b^ + 0.313	164.8 ^c^ + 0.484
Quercetin	14.45 ^a^ + 0.116	12.86 ^a^ + 0.088	10.89 ^a^ + 0.009	20.86 ^a^ + 1.427	12.46 ^a^ + 0.062	19.73 ^a^ + 0.093	17.78 ^b^ + 0.147	8.001 ^c^ + 0.043	27.50 ^a^ + 0.009	14.94 ^bc^ + 0.015	23.42 ^ab^ + 0.099	13.60 ^bc^ + 1.087	15.45 ^d^ + 0.062	17.51 ^cd^ + 0.017	21.56 ^bc^ + 0.141	22.55 ^b^ + 0.081	22.31 ^b^ + 0.239	32.94 ^a^ + 0.432
Quercetin-3-β-D-Glucoside	9.173 ^ab^ + 0.051	5.284 ^b^ + 0.010	3.028 ^b^ + 0.010	19.08 ^a^ + 1.584	8.594 ^ab^ + 0.044	14.97 ^ab^ + 0.074	14.42 ^ab^ + 0.115	4.885 ^c^ + 0.011	18.24 ^a^ + 0.054	6.348 ^c^ + 0.007	15.08 ^ab^ + 0.105	10.80 ^bc^ + 0.898	21.51 ^a^ + 0.018	10.27 ^e^ + 0.005	9.532 ^e^ + 0.002	18.35 ^b^ + 0.051	12.68 ^d^ + 0.073	15.03 ^c^ + 0.171
Quercitrin	<LOQ	n.d.	n.d.	n.d.	n.d.	n.d.	<LOQ	n.d.	4.039 ^a^ + 0.008	<LOQ	n.d.	n.d.	n.d.	n.d.	n.d.	n.d.	n.d.	n.d.
Rutin	<LOQ	<LOQ	n.d.	1.316 ^a^ + 0.083	<LOQ	0.871 ^b^ + 0.005	0.961 ^a^ + 0.003	<LOQ	1.177 ^a^ + 0.000	<LOQ	1.028 ^a^ + 0.005	0.974 ^a^ + 0.055	4.650 ^a^ + 0.012	<LOQ	0.766 ^c^ + 0.001	1.327 ^b^ + 0.003	0.901 ^c^ + 0.016	1.279 ^b^ + 0.015
Taxifolin	<LOQ	n.d.	n.d.	n.d.	n.d.	n.d.	n.d.	n.d.	<LOQ	n.d.	<LOQ	n.d.	n.d.	n.d.	n.d.	<LOQ	n.d.	<LOQ
Vanilic Acid	1.400 ^a^ + 0.014	<LOQ	1.731 ^a^ + 0.004	1.424 ^a^ + 0.064	<LOQ	<LOQ	n.d.	n.d.	n.d.	n.d.	n.d.	n.d.	<LOQ	<LOQ	<LOQ	<LOQ	1.432 ^a^ + 0.008	1.557 ^a^ + 0.009

**Table 3:** Physical and sensory characteristics of three apple cultivars (Gala Redlum, Granny Smith, and Fuji Aztec), including average size, weight, and °Brix. Notes highlight specific attributes related to flavor, texture, and common uses.

Cultivar	Average Size	Average Weight	Brix (°Bx)	Notes
Gala Redlum	65–70 mm in diameter	140–180 g	12–15 °Bx	Appreciated for its strong red coloration and typically sweet flavor. It maintains the crisp texture of standard Gala.
Granny Smith	70–80 mm in diameter	170–220 g	11–13 °Bx	Known for its tartness and firm texture. It generally has lower sugar than red cultivars, and is often used in cooking or for its contrast in fresh eating.
Fuji Aztec	70–80 mm in diameter	170–220 g	14–18 °Bx	Known for its dense, sweet flesh and can exceed typical Fuji Brix levels under good growing conditions.

## Data Availability

The raw data supporting the conclusions of this article will be made available by the authors on request.
